# Prenucleation Cluster Pathway is Inconsistent with CaCO_3_ Kinetics

**DOI:** 10.1021/acs.cgd.4c00092

**Published:** 2024-04-25

**Authors:** Robert Darkins, Dorothy M. Duffy, Ian J. Ford

**Affiliations:** Department of Physics and Astronomy, University College London, Gower Street, London WC1E 6BT, United Kingdom

## Abstract

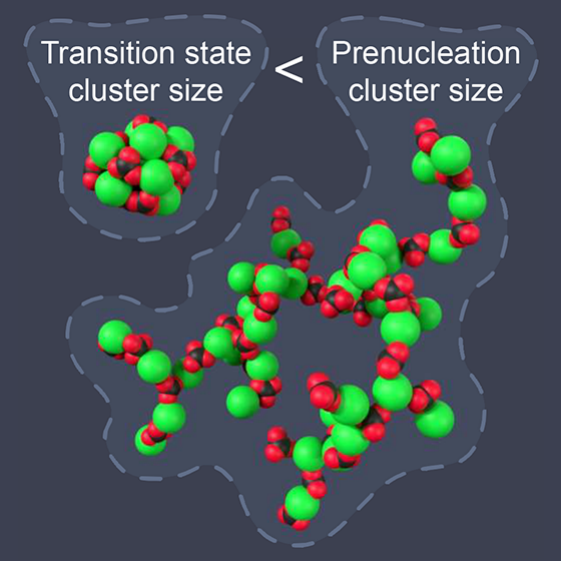

It has been debated whether CaCO_3_ nucleates classically with the attainment of a critical cluster size or nonclassically with the restructuring of a prenucleation cluster (PNC). Here, we determine from the nucleation kinetics of CaCO_3_ that the transition state is composed of about 10 formula units, irrespective of the supersaturation. Crucially, the size of the transition state is considerably smaller than the average PNC size estimated from experimental characterization. This size discrepancy suggests the PNCs are uninvolved in nucleation, and the kinetics indicate that if CaCO_3_ nucleates classically, the transition state must be an abnormally unstable (antimagic) cluster.

## Abstract 2

The nucleation mechanism of CaCO_3_ is elucidated by the transition state cluster size which we determine from the nucleation kinetics.

The principles of classical nucleation theory were challenged in 2008 when Gebauer et al.^1^ reported that aqueous CaCO_3_ solutions contain populations of clusters composed of dozens of formula units. These so-called prenucleation clusters (PNCs) emerge even below saturation, they lack a phase interface, their growth is bounded, and simulation^2^ and X-ray scattering^3,4^ suggest that they have chain-like structures. It has been argued that CaCO_3_ nucleates not when a critical cluster size is attained, as in classical nucleation theory, but when a PNC transforms from its chain-like configuration into a more compact structure with a phase interface.^5,6^ This nonclassical PNC pathway has been contested,^7−9^ although the arguments against it have not been unassailable.^5^

We determine here the number of formula units, n*, in the transition state cluster. We first establish that n* can be computed from the nucleation kinetics regardless of whether CaCO_3_ nucleates classically or nonclassically. We then obtain n* from existing experimental data and compare the results with the predictions of both classical nucleation theory and the nonclassical PNC pathway.

Central to our discussion is the first nucleation theorem,^10^ which relates n* to the nucleation kinetics:
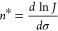
1where *J* is the nucleation rate, σ = ln(IAP/*K*_sp_) is the saturation index, IAP is the ion activity product, and *K*_sp_ is the solubility product of the nucleating phase. Equation 1 was originally derived by Kashchiev within the framework of classical nucleation theory. The PNC pathway differs from classical nucleation theory in that nonclassical nucleation is limited by an event orthogonal to the cluster size variable: it is limited by the structural transformation of a PNC. For this reason, the original derivation of eq 1 does not strictly translate to the PNC pathway. The derivation can nevertheless be adapted with minor alteration, as we now show.

Suppose that each cluster in the solution is specified by both its size *n* and an order parameter λ. The order parameter distinguishes the chain-like PNC structure from the nucleating phase. The work required to form a cluster (*n*, λ) in a solution with a saturation index σ can be written in the form
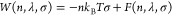
2where *k*_B_ is the Boltzmann constant, *T* is the temperature, and the excess free energy *F* defines the size distribution and stability of all clusters in the reaction space. We do not need to specify *F*.

Nucleation will be dominated by a particular pathway through the (*n*, λ) reaction space, and the transition state will correspond to the point (n*, λ*) along this pathway that maximizes *W* (Figure 1a). The nucleation rate will then take the form
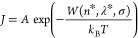
3where the pre-exponential factor *A* will be independent of σ, assuming as claimed^11^ that the PNCs are in equilibrium with the ions. Otherwise, *A* will have a near-linear dependence on exp(σ) due to the kinetics of cluster formation.^10^ Note that *A* implicitly captures all of the intricacies of barrier kinetics but that only its relationship to σ will be of consequence.

**Figure 1:**
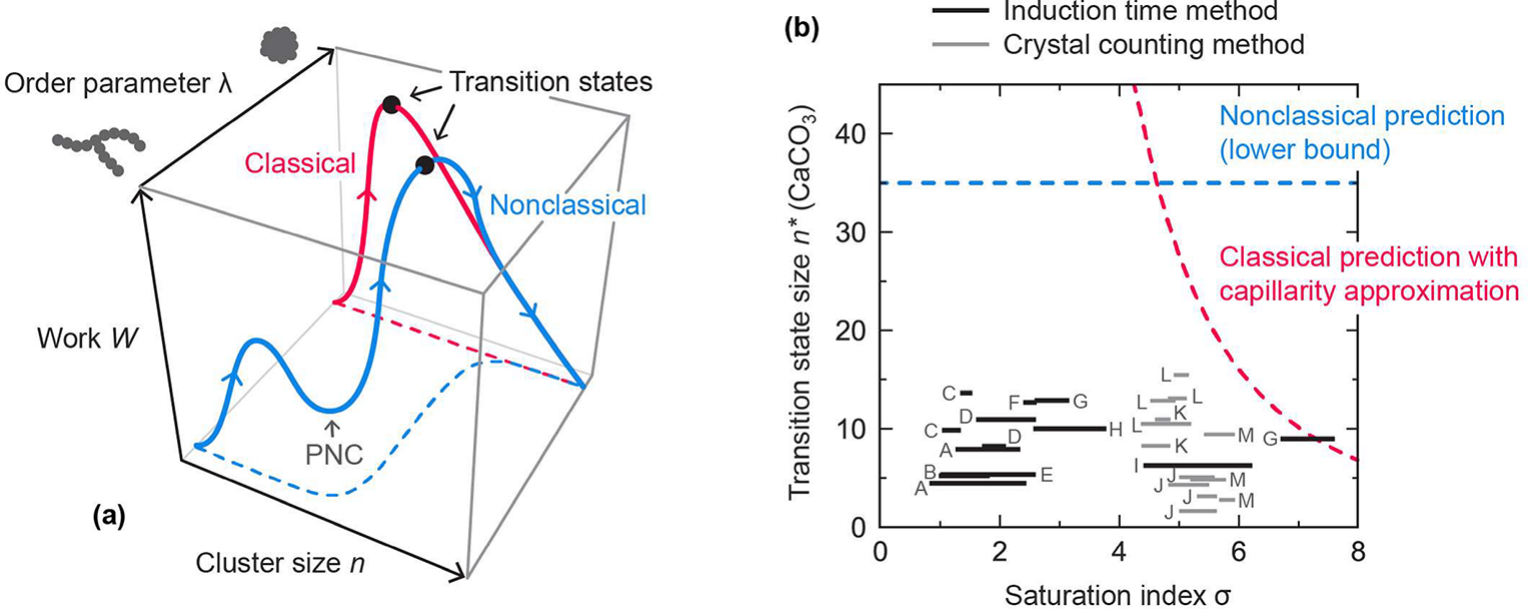
(a) Illustrative examples of classical and nonclassical nucleation pathways through the reaction space. The order parameter distinguishes the chain-like PNCs from the nucleating phase. (b) Solid lines show the transition state sizes in formula units determined from various nucleation experiments. Each line spans the σ values sampled in the experiment. Labels identify the sources: A,^12^ B,^13^ C,^14^ D,^15^ E,^16^ F,^17^ G,^18^ H,^19^ I,^20^ J,^21^ K,^22^ L,^23^ M.^24^ Dashed lines are theoretical predictions based on other experimental characterization. The nonclassical prediction is deemed to be a lower bound on n*.

Combining and rearranging eqs 2 and 3 gives
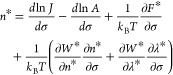
4where an asterisk denotes evaluation at the transition state, e.g., . A step-by-step derivation of eq 4 can be found in the Supporting Information. Given the description of *A* above, the second term on the right-hand side of eq 4 can be discarded with an error no greater than about one formula unit. The third term can also be discarded because the excess free energy *F* of a cluster in solution has a negligible dependence on σ.^10^ Finally, the fourth term is zero because the transition state corresponds to a saddle point of *W*,^25^ hence, ∂W*/∂n* = 0 and ∂W*/∂λ* = 0. In summary, eq 4 reduces to eq 1, and the first nucleation theorem applies to the PNC pathway. Our derivation may also apply to other nonclassical nucleation mechanisms, like the two-step pathway observed in metal clusters.^26,27^

(It so happens that the first nucleation theorem is violated by the only other quantitative PNC model that we know of. Specifically, a linear relationship has been derived between the concentration of PNCs and the concentration of ion pairs,^28^ which if true, would yield *d* ln *J*/*d*σ = 1 no matter the actual transition state size. This linear relationship, however, was erroneously derived. For example, in ref (28), equations S11 and S13 contradict each other and are in fact both wrong. The claimed result also violates the law of mass action.)

Using the first nucleation theorem, we have determined n* from published CaCO_3_ nucleation rates measured across a wide range of experimental conditions (Figure 1b). The collated data^12−24^ include the nucleation of both calcite^15−17,24^ and vaterite,^12,13,20^ heterogeneous nucleation on various organic substrates,^15,21−24^ purported homogeneous nucleation,^14,18^ pHs as low as 7^16^ and as high as 11,^21^ and supersaturations ranging from far below the solubility of amorphous calcium carbonate^12−14^ to far above it.^18^ These measurements can be divided into two types depending on the method used to establish *J*: either (1) the induction time *J*^–1^ was measured by detecting a change in pH, turbidity, etc.^12−20^ or (2) the number of crystals on a substrate, *Jt*, was counted as a function of time *t*.^21−24^

Interpreting the induction time measurements requires some care. In practice, the induction time *t*_*i*_ is the average time between the attainment of supersaturation and the detection of nucleation. Because the crystals must grow to a sufficient size to be registered, many nucleation events may occur before a single event is detected.^29^ If this is the case, and if the crystal size *L* increases over time according to a power law, *L* ∼ (*Gt*)^ν^, it can be shown that^30^
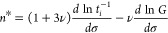
5where *G* defines the growth rate, and the growth exponent is ν ≈ 0.5 for CaCO_3_ crystals as small as 10 nm over the range σ ≳ 1 due to the limits of boundary layer diffusion.^31^ We determined n* using eq 5 for all of the induction time data. However, to avoid dispute over the form of *G*, we neglected the final term in eq 5 and therefore erred on the side of slightly overestimating n*.

Both the induction time and the crystal counting methods produced values of n* ranging from a few formula units up to 15, with an average value of about 10 formula units, and with no discernible dependence on σ. These results are surprising as they do not align with our cursory expectations based on either classical nucleation theory or the PNC pathway.

In classical nucleation theory, the function n*(σ) can be derived by assigning a spherical geometry and a macroscopic density and interfacial free energy to the clusters—i.e., by making the capillarity approximation. This approximation leads to the prediction that n* should increase dramatically as σ is decreased toward saturation, as illustrated in Figure 1b for an interfacial free energy of 120 mJ/m^2^.^32^ The lack of such a dependence between n* and σ in the kinetics-derived data rules out the capillarity approximation (successful applications^22−24^ of this approximation to CaCO_3_ have been confined to saturation ranges too narrow to expose its limitations). This does not rule out classical nucleation theory in general, however, as the independence between n* and σ could be attributed to a more complex excess free energy featuring an abnormally unstable cluster, that is, an antimagic cluster.^33^ We note that the thermodynamics of CaCO_3_ clusters computed using molecular simulation show no magic or antimagic clusters up to four formula units.^34^

If instead the PNC pathway is responsible for nucleation, then n* should be about the same size as the average PNC. (If n* were much larger than this, nucleation would be classical, and if n* were much smaller, the PNCs would implausibly have to decrease in size while crossing the nucleation barrier.) Small-angle X-ray scattering (SAXS) indicates that PNCs hardly change in size across the saturation range depicted in Figure 1b,^3^ consistent with the flat n*(σ) profile derived from the kinetics. However, in contrast to n* ≈ 10 formula units, the average PNC size has been estimated from analytical ultracentrifugation (AUC) to be *at least* 35 formula units.^11^ In support of this number, the PNC radius of gyration was determined using SAXS to be 3.5 nm under conditions (undersaturation and a low pH of 7.5) that are known to produce small PNCs.^3^ This radius of gyration would equate to about 20 formula units if the PNCs were perfectly straight chains, but the presence of branching and torsion, evidenced by the same scattering data, would significantly increase this size estimate.

Because the average PNC size determined from AUC and SAXS is considerably larger than the kinetics-derived n* (Figure 1b), we argue that either (1) the characterization methods are overestimating the average PNC size or (2) the PNCs are uninvolved in nucleation. In our view, the second option is likely even if the first option is also true. This is because some of the nucleation experiments exhibited transition state sizes as small as only a few formula units, which is difficult to reconcile with the PNC pathway in any case.

Turning to matters of polymorph selection, the transition state sizes reported here are probably too small to have crystalline polymorphs attributed to them.^35^ The polymorph must therefore be selected *after* nucleation, meaning that crystals with distinct polymorphs will arise from indistinguishable nucleation events and then diverge structurally during growth. Polymorph control should therefore not be interpreted in terms of competitive nucleation. This would explain why, in contrast to most inorganic materials, CaCO_3_ can form multiple polymorphs in a single reaction solution (e.g., ref (36)).

To conclude: the size of the transition state cluster for CaCO_3_ nucleation is readily determinable from the kinetics, and it provides a perspective that may well settle the mechanism debate. The evidence highlighted here supports a classical mechanism involving the creation of a critical antimagic cluster, with PNCs merely spectating the event.

## Supporting Information Available

Derivation of eq 4 (PDF)
