# Supplementary material for: Prenucleation Cluster Pathway is Inconsistent with CaCO3 Kinetics
Source: Cryst Growth Des. 2024 Apr 25;24(10):4013–6. doi: 10.1021/acs.cgd.4c00092 (PMC11099912; doi:10.1021/acs.cgd.4c00092)
Supplement: Supplementary file 1 — cg4c00092_si_001.pdf [file cg4c00092_si_001.pdf]

Supporting information for: Prenucleation cluster pathway is inconsistent with $CaCO_3$ kinetics

Robert Darkins,* Dorothy M. Duffy, and Ian J. Ford

Department of Physics and Astronomy, University College London, Gower Street, London, WC1E 6BT, UK

E-mail: r.darkins@ucl.ac.uk

Derivation of equation (4)

Here we elaborate on the steps required to derive equation (4) in the main text.

We begin by reproducing equations (2), (3) and (4) from the main text:

$$W(n, \lambda, \sigma) = -nk_BT\sigma + F(n, \lambda, \sigma) \tag{2}$$

$$J = A\exp\left(-\frac{W(n^*, \lambda^*, \sigma)}{k_BT}\right) \tag{3}$$

$$n^* = \frac{d\ln J}{d\sigma} - \frac{d\ln A}{d\sigma} + \frac{1}{k_BT}\frac{\partial F^*}{\partial\sigma} + \frac{1}{k_BT}\left(\frac{\partial W^*}{\partial n^*}\frac{\partial n^*}{\partial\sigma} + \frac{\partial W^*}{\partial\lambda^*}\frac{\partial\lambda^*}{\partial\sigma}\right) \tag{4}$$

Taking the logarithm of equation (3) and differentiating with respect to σ gives

$$\frac{d\ln J}{d\sigma} = \frac{d\ln A}{d\sigma} - \frac{1}{k_BT}\left.\frac{dW}{d\sigma}\right|_{(n^*,\lambda^*,\sigma)} \tag{S1}$$

$$= \frac{d\ln A}{d\sigma} - \frac{1}{k_BT}\left.\left(\frac{\partial W}{\partial\sigma} + \frac{\partial W}{\partial n}\frac{dn}{d\sigma} + \frac{\partial W}{\partial\lambda}\frac{d\lambda}{d\sigma}\right)\right|_{(n^*,\lambda^*,\sigma)} \tag{S2}$$

$$= \frac{d\ln A}{d\sigma} - \frac{1}{k_BT}\left.\left(-nk_BT + \frac{\partial F}{\partial\sigma} + \frac{\partial W}{\partial n}\frac{dn}{d\sigma} + \frac{\partial W}{\partial\lambda}\frac{d\lambda}{d\sigma}\right)\right|_{(n^*,\lambda^*,\sigma)} \tag{S3}$$

where equation (S2) follows from the chain rule, and equation (S3) is obtained by using equation (2) to evaluate the partial derivative $\frac{\partial W}{\partial\sigma}$.

Equation (S3) rearranges to

$$n^* = \frac{d\ln J}{d\sigma} - \frac{d\ln A}{d\sigma} + \frac{1}{k_BT}\left.\frac{\partial F}{\partial\sigma}\right|_{(n^*,\lambda^*,\sigma)} + \frac{1}{k_BT}\left.\left(\frac{\partial W}{\partial n}\frac{\partial n}{\partial\sigma} + \frac{\partial W}{\partial\lambda}\frac{\partial\lambda}{\partial\sigma}\right)\right|_{(n^*,\lambda^*,\sigma)} \tag{S4}$$

which can be reexpressed as equation (4) using the shorthand $\frac{\partial W^*}{\partial n^*} \equiv \frac{\partial W}{\partial n}(n^*, \lambda^*, \sigma)$, etc.
